# UDC-SNN: An Uncertainty-Aware Dynamic Cascading Framework with Spiking Neural Network for Balancing Performance and Energy in Multimodal Emotion Recognition

**DOI:** 10.3390/s26092859

**Published:** 2026-05-03

**Authors:** Guihao Ran, Shengzhe Li, Zhiwen Jiang, Han Zhang, Xinyuan Long, Dakun Lai

**Affiliations:** 1 School of Electronic Science and Engineering, University of Electronic Science and Technology of China, Chengdu 611731, China; 2 Haihe Laboratory for Brain-Computer Interaction and Human-Computer Collaboration, Tianjin 300000, China

**Keywords:** uncertainty-aware, spiking neural network, multimodal emotion recognition, energy efficiency

## Abstract

The aim of this study is to propose an uncertainty-aware dynamic cascading framework based on spiking neural network (UDC-SNN) for multimodal emotion recognition, particularly to address the inherent trade-off between recognition performance and energy efficiency. An asymmetric dynamic routing mechanism was proposed to enable demand-driven activation of the high-power electroencephalogram (EEG) branch, coupled with preliminary inference on a low-power electrocardiogram (ECG) branch and uncertainty quantification via Shannon entropy. Meanwhile, a parameter-free log-linear aggregation strategy was developed to transform modality-specific entropy into dynamic Bayesian weights through an exponential decay function, effectively mitigating the negative transfer effects induced by unimodal noise. The UDC-SNN was evaluated on the multimodal affective dataset DREAMER, comprising 23 subjects (170,660 segments). The averaged recognition accuracy and energy consumption across the three dimensions of valence, arousal, and dominance were 90.75% and 4.62 μJ, respectively. The obtained results suggest that the proposed framework could potentially achieve a favorable balance between high emotion recognition and low energy consumption, thereby establishing its applicability for real-time monitoring in resource-constrained scenarios.

## 1. Introduction

Emotional states constitute a central component of human cognition, behavioral decision-making, and social interaction, exerting profound influences on mental health and daily functioning [1,2,3,4]. Accurate emotion recognition holds considerable potential in domains such as intelligent healthcare, mental health monitoring, educational assistance, and brain–computer interfaces [5,6,7,8]. It may also contribute to improving human–computer interaction experiences and provide quantitative indicators for the auxiliary diagnosis of psychiatric disorders [5,9]. Traditional approaches based on observable behavioral cues, such as facial expressions and vocal prosody, can be effective in certain contexts; however, they are often susceptible to intentional masking, cultural variability, and environmental noise, thereby limiting their ability to comprehensively reflect intrinsic emotional states [10,11,12,13]. Consequently, the use of physiological signals for emotion recognition has emerged as a prominent research direction in affective computing and neural engineering [9,10,14].

Among various physiological signal modalities, electroencephalography (EEG) and electrocardiography (ECG) have attracted extensive attention due to their capability to reflect emotion-related physiological activities at different levels [15]. EEG records the collective activity of cortical neuronal populations with relatively high temporal resolution and can, to some extent, capture the dynamic changes of the central nervous system during emotional processing, thus holding particular significance in studies of emotion-related neural activity [16]. In contrast, ECG primarily reflects the regulatory state of the autonomic nervous system, where variations in cardiac rhythms can characterize emotion-elicited physiological responses from a peripheral perspective [10,17]. Furthermore, compared to single-modality approaches, multimodal fusion of EEG and ECG offers the potential to simultaneously capture the dynamic interactions between the central nervous system and the peripheral autonomic system. By integrating neural activity at the brain level with peripheral physiological responses, a more comprehensive representation of emotion-related physiological processes can be obtained, thereby providing richer and more reliable support for intelligent emotion recognition [15].

In the field of emotion recognition, a wide range of artificial intelligence models have progressively advanced diagnostic performance and practical applicability through methodological innovations. From the perspective of traditional machine learning, Sharma et al. (2021) [18] proposed an automatic emotion recognition model based on sliding mode singular spectrum analysis (SM-SSA), which decomposes physiological signals into reconstructed components (RCs) and extracts information potential (IP) and centered correntropy (CEC) features, achieving classification accuracies of 92.06% for arousal, 92.38% for dominance, and 92.30% for valence on the DREAMER dataset. In 2022, Nalwaya et al. [19] employed a Fourier–Bessel series expansion-based empirical wavelet transform (FBSE-EWT) to decompose EEG and ECG signals into multiple sub-bands, followed by the computation of spectral entropy features; using a K-nearest neighbors (KNN) classifier, they reported accuracies of 97.84% for arousal, 97.91% for valence, and 97.86% for dominance on the same dataset.

With respect to conventional artificial neural networks, researchers have focused on developing end-to-end deep models for automatic feature extraction. In 2022, Siddharth et al. [20] utilized a deep convolutional neural network (CNN) to extract VGG-based features from EEG topographic maps and ECG spectrograms, achieving an accuracy of 79.95% for both valence and arousal in multimodal recognition on the DREAMER dataset. In 2023, Khan et al. [21] proposed an attention-based two-layer gated recurrent unit model (AT2GRU), incorporating a maximum discrepancy-based heterogeneous mitigation (MDHM) method to alleviate device-related variability, and reported classification accuracies of 84.15% for valence, 84.69% for arousal, and 85.81% for dominance. More recently, in 2025, Wang et al. [22] designed a hybrid Att-1DCNN-GRU network, where physiological signal features were selected using a random forest algorithm, achieving accuracies of 95.95%, 94.93%, and 94.91% for valence, arousal, and dominance, respectively, on the DREAMER dataset.

In summary, although the aforementioned methods have demonstrated notable progress in emotion recognition, several limitations remain. Traditional machine learning approaches offer advantages such as conceptual simplicity and interpretability; however, their performance is highly dependent on handcrafted feature design and may exhibit limited generalization capability. Conventional artificial neural networks (ANNs) enable automatic feature extraction, yet many studies primarily emphasize performance improvements while relatively overlooking energy efficiency. Therefore, there is a need to explore an end-to-end emotion recognition framework that can simultaneously maintain high performance and energy efficiency.

Motivated by these considerations, this study proposes an uncertainty-aware dynamic cascading framework based on spiking neural networks (UDC-SNN) for emotion recognition. Specifically, the proposed method introduces a dynamic routing mechanism with asymmetric computational cost: a low-power ECG modality is preferentially employed for preliminary inference, while decision uncertainty is quantified in real time via Shannon entropy. The high-power EEG modality is activated on demand only when a sample is identified as highly uncertain. In the multimodal decision stage, instead of conventional static (fully connected layers) black-box fusion, a parameter-free log-linear pooling strategy is introduced. Practically, modality-wise entropy is transformed into dynamic Bayesian weights through an exponential decay function, thereby adaptively mitigating negative transfer caused by transient unimodal noise at a principled mathematical level. Overall, this work is expected to provide an effective paradigm for balancing performance and energy consumption, offering a potentially valuable reference for real-time affective computing in resource-constrained environments.

## 2. Materials and Methods

### 2.1. Framework

**Figure 1:**
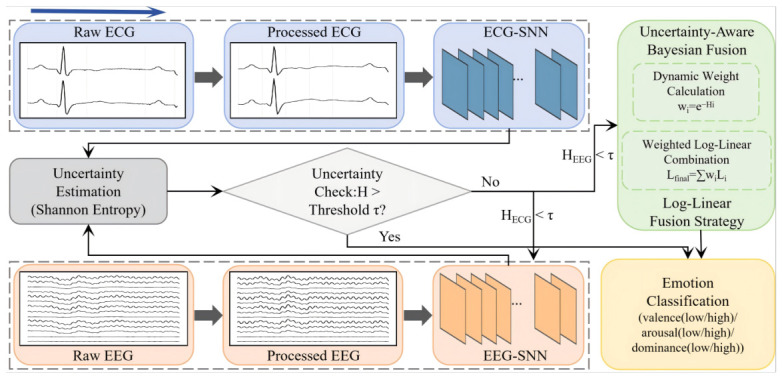
The framework of the system.

Figure 1 illustrates the proposed framework for automatic emotion recognition. The framework is constructed based on SNN and incorporates a multi-stage uncertainty-aware dynamic cascading strategy to perform binary classification of emotional valence, arousal, and dominance levels. The overall pipeline consists of three core stages: signal preprocessing, SNN-based feature extraction model training, and adaptive cascading inference and decision-making. The blue arrows in the figure represent the operational flow of the system.

### 2.2. Datasets

This study utilizes the DREAMER multimodal affective dataset, which was released by the University of the West of Scotland. The dataset comprises physiological responses from 23 healthy participants while watching 18 emotion-eliciting video clips. After viewing each clip, participants provided self-assessment ratings for valence, arousal, and dominance on a scale from 1 to 5.

In terms of signal acquisition, both EEG and ECG signals were synchronously recorded. The EEG signals were collected using the Emotiv EPOC wireless device with 14 channels at a sampling rate of 128 Hz, while the ECG signals were acquired using SHIMMER sensors in a dual-channel configuration at 256 Hz. To mitigate the effects of inter-subject variability and environmental noise, a baseline recording phase was included prior to each stimulus video, which was later used for signal calibration and normalization.

To ensure signal quality and the reliability of model evaluation, a systematic preprocessing pipeline was applied to the DREAMER dataset. First, a 5th-order Butterworth bandpass filter was employed to denoise the EEG (4–45 Hz) and ECG (0.5–45 Hz) signals. The ECG signals were subsequently downsampled to 128 Hz to achieve temporal alignment across modalities. Next, the last 4 s of the pre-stimulus baseline segment were extracted to compute the mean amplitude, which was then subtracted from the corresponding stimulus-evoked signals to perform baseline correction, thereby reducing inter-subject physiological variability.

For label processing, the continuous self-assessment scores (ranging from 1 to 5) for valence, arousal, and dominance were binarized using a threshold of 3.0, where values greater than 3.0 were mapped to the high-level class and values less than or equal to 3.0 to the low-level class [19,21]. To augment the dataset while preserving temporal dynamics, a sliding window approach was adopted, with a window length of 1 s (128 sampling points) and a 50% overlap, to segment the continuous signals.

During dataset partitioning, a stratified split was performed based on the joint labels formed by combining the three emotional dimensions, ensuring consistency in the overall class distribution. The dataset was strictly divided into training, validation, and test sets in a ratio of 8:1:1. Subsequently, channel-wise Z-score normalization was applied to the segmented data. Importantly, the normalization parameters were computed exclusively on the training set and then applied to the entire dataset, effectively eliminating scale discrepancies while preventing potential data leakage.

Following preprocessing, a total of 170,660 multimodal samples were obtained, as summarized in Table 1. Based on the binarized labels, the proportion of samples categorized as “high” in the valence, arousal, and dominance dimensions is 41.1%, 47.9%, and 53.7%, respectively. This relatively large-scale and distribution-balanced dataset provides a solid foundation for the effective training of deep learning models.

### 2.3. Uncertainty-Aware Dynamic Cascading Framework Based on Spiking Neural Network

#### 2.3.1. Uncertainty-Aware Dynamic Cascading Strategy

Motivated by the asymmetric computational cost of physiological signals—where the 14-channel EEG is intensive, whereas the 2-channel ECG is more lightweight—the proposed method establishes an uncertainty-aware dynamic cascading strategy. Specifically, an on-demand dynamic routing mechanism is adopted and integrated with Bayesian decision fusion.

More concretely, the inference procedure is formulated as a three-stage cascading architecture. For a given input, the system first activates the low-cost ECG spiking network to extract preliminary features and generate unnormalized log-probabilities (i.e., logits), denoted as $L_{ecg}\in R^{C}$, where *C* represents the number of emotion classes. If the maximum predicted probability of the ECG modality exceeds a predefined threshold τ, an early-exit mechanism is triggered. At the physical level, this effectively blocks the input from entering the EEG pathway, thereby ensuring zero spike activity within the EEG network.

When the ECG modality exhibits high uncertainty (i.e., confidence ≤τ), the system dynamically activates the EEG network to capture neural features, producing $L_{eeg}$. Similarly, if the maximum predicted probability of the EEG modality exceeds the threshold τ, an early exit is performed. Otherwise, when the EEG modality still reflects high uncertainty, the system proceeds to the final multimodal fusion stage.

From a probabilistic perspective, multimodal decision-making can be formulated as a maximum a posteriori (MAP) estimation problem. Assuming conditional independence between EEG and ECG modalities given the emotional state *Y*, the joint posterior can be expressed, according to Bayes’ theorem, as

(1) $$P\left(Y|X_{eeg},X_{ecg}\right)\propto P\left(Y|X_{eeg}\right)\cdot P\left(Y|X_{ecg}\right)$$

To simplify computation, the multiplicative formulation is transformed into an additive aggregation of evidence via a logarithmic transformation. Since the logits produced by neural networks can be interpreted as unnormalized log-posterior probabilities, Bayesian fusion naturally reduces to element-wise addition:

(2) $$\log P\left(Y|X_{eeg},X_{ecg}\right)\propto L_{eeg}+L_{ecg}$$

However, the naive Bayesian assumption implicitly assigns equal confidence to both modalities, which may be suboptimal in the presence of transient physiological artifacts. To address this limitation, Shannon entropy is employed to independently quantify the epistemic uncertainty of each modality:

(3) $$H_{m}=-\sum_{i=1}^{C}p_{m,i}\log p_{m,i},\;m\in \left\{eeg,ecg\right\}$$

where $p_{m,i}$ denotes the predicted probability of modality *m* for class *i*. A higher entropy value $H_{m}$ corresponds to a flatter probability distribution, indicating greater model uncertainty and increased susceptibility to noise.

To dynamically calibrate the contribution of each modality, an exponentially decaying function, inspired by statistical mechanics, is introduced to model reliability weights:

(4) $$w_{m}=\exp^{\left(-H_{m}\right)}$$

This transformation ensures that modalities with high uncertainty are strongly down-weighted ($w_{m}\to 0$), while more confident modalities exert a dominant influence on the decision boundary. After normalization via $w_{m}^{\prime }=\frac{w_{m}}{w_{eeg}+w_{ecg}}$, the final joint decision is obtained through log-linear fusion:

(5) $$L_{\mathrm{final}}=w_{eeg}L_{eeg}+w_{ecg}L_{ecg}$$

The final emotion class is determined by $\arg\max(L_{final})$. By projecting modality-specific features into the log-probability space, the framework is able to adaptively suppress modality-specific interference without introducing additional trainable parameters, thereby maintaining both computational efficiency and modeling interpretability.

#### 2.3.2. Spiking Convolutional Neural Network (SCNN)

**Figure 2:**
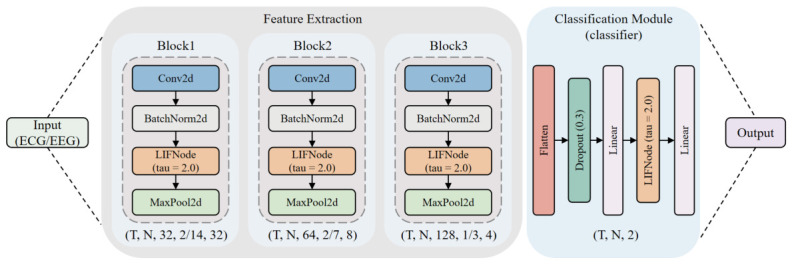
The architecture of the model.

In this study, a unified and lightweight spiking convolutional neural network (SCNN) architecture is developed, as illustrated in Figure 2. The model consists of three progressive feature extraction stages and a two-layer classifier, collaboratively capturing spatiotemporal characteristics of physiological signals. The core design principle lies in a hierarchical feature extraction strategy: the first two stages (Stage 1 and Stage 2) employ asymmetric convolutional kernels (with sizes of (3,7) and (3,5), respectively) to achieve substantial temporal downsampling while preserving spatial resolution, whereas the third stage utilizes standard 3×3 convolutions to facilitate deeper feature integration.

Each stage follows a pipeline structure of “convolution–normalization–spike generation–pooling.” Within this pipeline, the Leaky Integrate-and-Fire (LIF) neuron (with a membrane time constant τ=2.0) serves as the nonlinear activation unit, emulating the dynamic spiking behavior of biological neurons and enabling nonlinear feature representation. To facilitate efficient training, the ArcTangent (ATan) function is adopted as a surrogate gradient for backpropagation.

Considering the substantial differences in spatial dimensionality (i.e., number of electrodes) between EEG and ECG signals, modality-specific adaptations are introduced in Stage 2. For EEG, full spatial downsampling is applied to reduce inter-electrode redundancy, whereas for ECG, the spatial dimension is preserved to prevent premature loss of information from its dual-channel configuration.

The classification module consists of two fully connected layers, with a Dropout mechanism (p=0.3) incorporated between them to mitigate overfitting. Additionally, LIF neurons are integrated within the classifier to provide nonlinear transformation.

The main parameter settings of the final model are shown in Table 2. To account for the characteristics of different modalities (ECG/EEG), targeted adjustments were made primarily to the pooling stride in Stage 2 and the label smoothing coefficients.

In the spiking neuron module of the model, the study employs the Leaky Integrate-and-Fire Node (LIFNode) neurons with an ATan surrogate gradient function.

The subthreshold neural dynamics of the LIFNode can be expressed as follows [23]:

(6) $$H\left[t\right]=V\left[t-1\right]+\frac{1}{\tau }\left(X\left[t\right]-\left(V\left[t-1\right]-V_{\mathrm{reset}}\right)\right)$$

where V[t−1] denotes the membrane potential at the previous time step, τ is the membrane time constant, X[t] represents the input signal, $V_{\mathrm{reset}}$ is the reset potential to which the membrane voltage returns after spike firing, and H[t] is the membrane potential at the current time step.

The ATan surrogate gradient function is defined as follows [23]:

(7) $$g\left(x\right)=\frac{1}{\pi }\arctan\left(\frac{\pi }{2}\alpha x\right)+\frac{1}{2}$$

where *x* denotes the input to the neuronal membrane potential, and α is a scaling factor that controls the slope and smoothness of the surrogate gradient function.

## 3. Experiment

### 3.1. Experimental Setup

In the present experiments, the time step (T) of the SNN is set to 4, aiming to achieve a balance between computational cost and feature modeling capacity. The model is optimized using the AdamW optimizer, with an initial learning rate of 0.01. A cosine annealing schedule is employed to dynamically adjust the learning rate, which may facilitate faster convergence while reducing the likelihood of being trapped in local minima. The training process is conducted for up to 200 epochs, with an early stopping strategy applied if the validation loss does not improve for 30 consecutive epochs, thereby mitigating the risk of overfitting.

In this study, SpikingJelly (0.0.0.0.14) is adopted as the implementation framework for SNN [23]. Built upon PyTorch, SpikingJelly provides an integrated platform for the development, training, and evaluation of SNN models. It supports flexible construction of multi-layer spiking architectures and offers functionalities including neuromorphic data processing, network design, and surrogate gradient-based optimization.

### 3.2. Evaluation Metrics

#### 3.2.1. Classification Performance Metrics

To comprehensively evaluate the classification performance of the proposed system, several standard metrics are employed, including Accuracy (Acc), Recall (Rec), Specificity (Spe), Precision (Pre), F1-score (F1), and the Area Under the Receiver Operating Characteristic Curve (AUC). These metrics provide a multi-dimensional assessment of classification performance. Their formulas are as follows:

(8) $$\mathrm{Acc}=\frac{TP+TN}{TP+TN+FP+FN}$$

(9) $$\mathrm{Rec}=\frac{TP}{TP+FN}$$

(10) $$\mathrm{Spe}=\frac{TN}{TN+FP}$$

(11) $$\mathrm{Pre}=\frac{TP}{TP+FP}$$

(12) $$F1=\frac{2TP}{2TP+FP+FN}$$

where TP, TN, FP, and FN denote the numbers of True Positives, True Negatives, False Positives, and False Negatives, respectively.

#### 3.2.2. Energy Consumption Metrics

Given that the model is based on SNN, the total number of operations can be defined as follows [24,25]:

(13) $$\mathrm{Total}\;OP_{SNN}=\mathrm{Overall}\;\mathrm{Spike}\;\mathrm{Rate}\times \mathrm{Total}\;OP_{ANN}$$

(14) $$\mathrm{Overall}\;\mathrm{Spike}\;\mathrm{Rate}=\frac{\mathrm{Total}\;\mathrm{Spikes}(\mathrm{all}\;\mathrm{layers}\;\mathrm{and}\;\mathrm{all}\;T)}{\mathrm{Total}\;\mathrm{Neurons}}$$

where the Overall Spike Rate is the total number of spikes across all layers and time steps divided by the total number of neurons. Total $OP_{ANN}$ refers to the number of operations in an ANN with the same architecture as the SNN.

Furthermore, compared to conventional ANNs, SNNs exhibit potentially higher energy efficiency and improved biological interpretability. Traditional ANNs rely on continuous multiply–accumulate (MAC) operations, whereas SNNs operate via discrete spike-driven mechanisms, where neurons emit spikes only when their membrane potentials exceed a threshold. This event-driven paradigm reduces redundant computations, as each operation typically involves only addition (ADD). Accordingly, the energy consumption of ANN and SNN models can be expressed as follows [24,25]:

(15) $${\mathrm{Energy}}_{ANN}=\mathrm{Total}\;OP_{ANN}\times {\mathrm{Energy}}_{MAC}$$

(16) $${\mathrm{Energy}}_{SNN}=\mathrm{Total}\;OP_{SNN}\times {\mathrm{Energy}}_{ADD}$$

wherein, ${Energy}_{MAC}$ and ${Energy}_{ADD}$ denote the energy consumption of MAC and ADD operations, respectively.

## 4. Results and Discussion

### 4.1. Classification Performance

#### 4.1.1. Analysis of Basic ECG/EEG Models (Classification Performance)

In this study, the classification performance of ECG and EEG models is first evaluated separately across different emotional labels. For each label category, corresponding unimodal models are independently trained. The experimental results (as shown in Table 3) indicate that the EEG-based models achieve accuracy values exceeding 88% across all labels, demonstrating consistently superior performance compared to the ECG-based models. This advantage may be attributed to the richer spatiotemporal information embedded in multi-channel EEG signals. Among the evaluated dimensions, the EEG model achieves its best performance on the dominance label, with an accuracy of 89.10%.

Furthermore, the impact of the temporal simulation length (Time Step, *T*) in spiking neural networks on model performance is investigated. Specifically, classification results under T=3,4, and 5 are examined (as shown in Table 4 and Table 5).

The results suggest that, for the ECG model (Table 4), the highest accuracies for valence, arousal, and dominance are achieved at T=4 (81.41%), T=5 (81.68%), and T=5 (83.56%), respectively. In contrast, for the EEG model (Table 5), the optimal performance across all three dimensions is consistently obtained at T=4, with accuracies of 88.06%, 88.36%, and 89.10%, respectively. It is worth noting that although the ECG model attains its peak accuracy at T=5, the improvement over T=4 (arousal: 81.58%, dominance: 83.14%) remains relatively marginal.

A comprehensive analysis indicates that models with T=3 generally exhibit lower accuracy, which may be due to the limited time step constraining the model’s ability to capture complex temporal dynamics. Conversely, in some cases, performance degradation observed at T=5 may be attributed to the introduction of redundant information or increased susceptibility to stochastic noise under longer temporal windows. These observations suggest that the adopted parameter configuration is reasonably.

Moreover, even under its optimal configuration (e.g., 83.56% for dominance at T=5), the ECG model does not surpass the lower-bound performance of the EEG model under minimal temporal settings (e.g., 85.77% for dominance at T=3). This further indicates that the EEG model demonstrates relatively stronger robustness and overall performance.

#### 4.1.2. Analysis of UDC-SNN (Classification Performance)

Subsequently, the overall performance of the proposed UDC-SNN framework is evaluated. As presented in Table 6, the framework achieves accuracy values exceeding 90% across all emotional dimensions, with the highest accuracy observed in the dominance dimension (91.07%). Comparative analysis shows that UDC-SNN consistently outperforms the original unimodal ECG and EEG models across all evaluation metrics.

**Figure 3:**
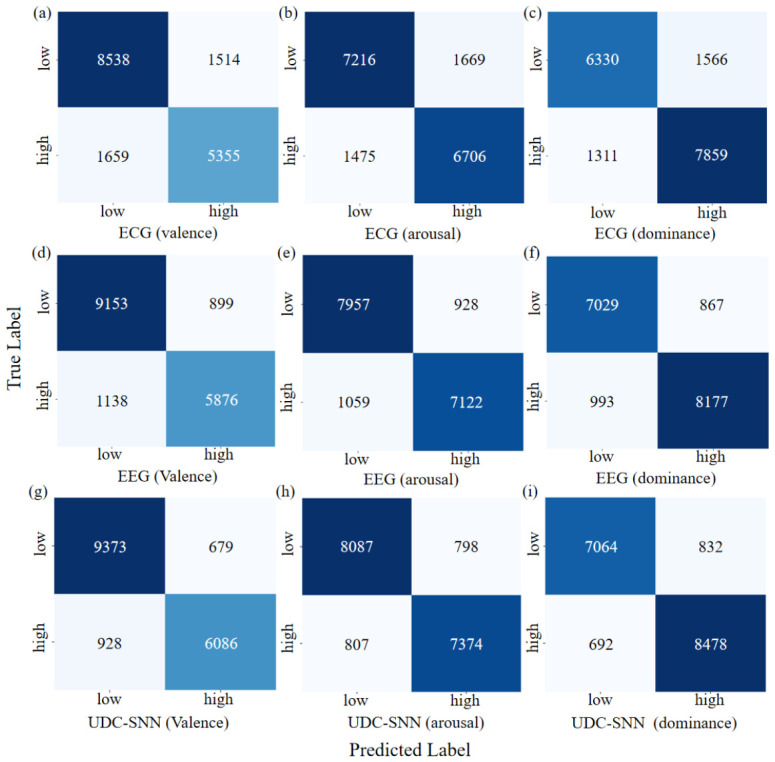
Comparison of confusion matrices between UDC-SNN and ECG/EEG baseline models across different dimensions. (**a**–**c**) The confusion matrices of the ECG-based model for the valence, arousal, and dominance dimensions, respectively; (**d**–**f**) those of the EEG-based model for the same three dimensions; and (**g**–**i**) the confusion matrices of UDC-SNN for valence, arousal, and dominance, respectively.

Figure 3 further illustrates the confusion matrices of UDC-SNN and baseline models across different emotional dimensions. The analysis reveals that, although the EEG model generally yields a higher number of true positives across categories compared to the ECG model, the proposed framework achieves further improvements in classification accuracy for all classes. This suggests that UDC-SNN effectively integrates heterogeneous modality information, thereby reducing classification ambiguity and exhibiting enhanced predictive consistency and robustness in multidimensional emotion recognition tasks.

Furthermore, to provide an additional assessment of the robustness of the proposed UDC-SNN framework, an extra emotional dataset, AMIGOS, was incorporated for evaluation. Specifically, following a transfer learning paradigm, only a small proportion of samples from the new dataset (≤10%) was used to fine-tune the pre-trained baseline model (ECG, EEG). This setting was intended to simulate a scenario in which both modalities exhibit relatively limited performance. Under this condition, the proposed framework was subsequently applied to examine its potential effectiveness.

For the AMIGOS dataset, the data were partitioned into training, validation, and test sets with a ratio of 1:2:7, respectively, where a relatively large test set was adopted to ensure a more adequate evaluation. The fine-tuning procedure was conducted for up to 30 epochs, with an early stopping strategy of 5 epochs, and a learning rate of 0.0001.

The final results are presented in Table 7. It can be observed that when both ECG and EEG modalities are in comparatively suboptimal conditions (ECG: valence: 68.49%; arousal: 57.89%; dominance: 62.85%. EEG: valence: 66.07%,; arousal: 61.36%; dominance: 64.65%), the proposed framework still able to contribute to the final recognition performance (valence: 71.16%; arousal: 63.31%; dominance: 68.68%). These findings suggest that the proposed framework is capable of maintaining effectiveness across varying levels of modality performance, thereby indicating its robustness.

### 4.2. Energy Consumption

#### 4.2.1. Analysis of Basic ECG/EEG Models (Energy Consumption)

**Figure 4:**
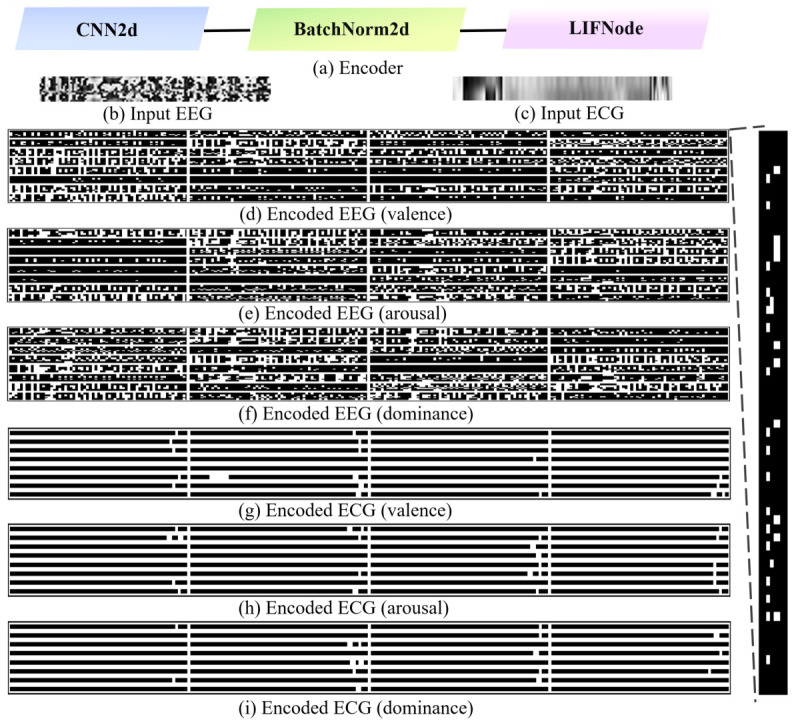
Schematic diagram of model encoding. (**a**) Architecture of the encoder module; (**b**) EEG input signal; (**c**) ECG input signal; (**d**–**f**) 32-channel spike outputs of EEG signals at the final time step across the three affective dimensions (valence, arousal, and dominance); (**g**–**i**) 32-channel spike outputs of ECG signals at the final time step across the same three dimensions (white dots represent spikes).

This section first evaluates the energy consumption characteristics of the ECG and EEG models across different emotional labels. The spike encoding directly determines the subsequent spiking rate within the network. The corresponding results are illustrated in Figure 4. Specifically, Figure 4a presents the end-to-end encoder architecture adopted in this study, which consists of the first three layers of the model (i.e., convolution, normalization, and LIF neuron layers). This module primarily converts continuous analog signals into spike trains via LIF neurons.

Figure 4b,c depicts the raw EEG and ECG signals prior to encoding, respectively. Figure 4d–i illustrates the encoded spike distributions of EEG and ECG signals across the three emotional dimensions. All spike visualizations correspond to the outputs at the final time step, where white pixels indicate neuronal spike firing events.

**Figure 5:**
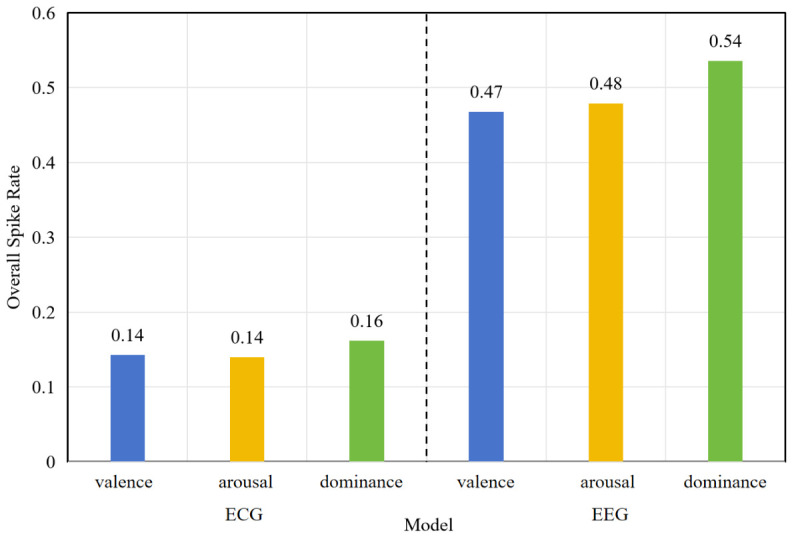
The overall spike rate of the ECG and EEG models under different labels.

Subsequently, the spiking rates of the ECG and EEG models are quantitatively evaluated by inputting a sample for each emotional dimension. As shown in Figure 5, the overall spiking rates of the ECG model remain relatively low across the three dimensions, with values of 0.14, 0.14, and 0.16, respectively. In contrast, the EEG model exhibits comparatively higher spiking rates, measured at 0.47, 0.48, and 0.54. These quantitative findings are consistent with the spike encoding patterns observed in Figure 4. Due to the limited number of electrode channels in ECG signals, the corresponding feature information density is relatively constrained, resulting in sparser spike representations after encoding. Although the EEG model demonstrates more frequent spiking activity, its overall firing rate remains within a reasonable range for efficient SNN computation.

Overall, the spiking rate serves as a key indicator of energy efficiency in SNNs, directly influencing the extent to which computational overhead can be reduced relative to conventional ANNs. Lower spiking rates imply a more pronounced advantage of event-driven computation, enabling a substantial reduction in effective operations during inference and, consequently, lower energy consumption.

To further quantify energy efficiency, this study conducts a systematic comparison between SNN and ANN under a 45 nm CMOS technology. As shown in Table 8, under 32-bit precision, the energy consumption of a single multiply–accumulate (MAC) operation in ANN is approximately 4.6 pJ, whereas a basic addition (ADD) operation in SNN requires only 0.9 pJ, corresponding to an approximate 5.1× difference in energy cost [26]. Although these values may vary with fabrication processes, it is generally observed that, within mainstream CMOS logic, adders incur significantly lower energy consumption than multipliers.

Based on Equations (13)–(16), together with the parameters listed in Table 8, a normalized energy analysis is conducted for ECG and EEG models across different emotional dimensions. As summarized in Table 9, with the energy consumption of the corresponding ANN models normalized to 1, the SNN-based models consistently demonstrate substantial energy efficiency gains. In particular, the ECG-valence and ECG-arousal models consume only approximately 7% of the energy required by their ANN counterparts, corresponding to an improvement of about 15.08× in energy efficiency. Even for the EEG-dominance task, which exhibits a relatively higher spiking rate, the energy consumption remains around 16% of the ANN baseline, yielding an approximate 6.28× improvement. These results highlight the advantages of SNNs in low-power computation, primarily due to their event-driven and sparsity-aware characteristics.

Furthermore, a detailed quantitative evaluation of energy consumption across different models and emotional labels is conducted, as reported in Table 10. The results indicate that the proposed models maintain relatively low parameter counts, computational complexity, and energy consumption, thereby exhibiting a lightweight design. Specifically, the ECG-based models contain approximately 0.24 M parameters, while the EEG-based models comprise around 0.5 M parameters. In terms of computational cost, the ECG-valence and ECG-arousal models require only 0.52 M floating-point operations (FLOPs) and incur an inference energy consumption of approximately 0.73 μJ. Even for the more computationally demanding EEG-dominance task, the FLOPs and energy consumption are controlled at approximately 7.66 million and 9.07 μJ, respectively.

#### 4.2.2. Analysis of UDC-SNN (Energy Consumption)

This section provides a quantitative evaluation of the energy efficiency of the proposed UDC-SNN framework, with the experimental results summarized in Table 11. The last two columns of the table report the computational complexity (FLOPs) and energy consumption of a conventional ECG–EEG sequential fusion scheme, which serves as the baseline. The results indicate that, owing to the on-demand dynamic routing mechanism, more than 50% of samples across all emotional dimensions can be classified at the first stage (i.e., the low-power ECG-based preliminary model), thereby enabling early exit. Among the three dimensions, the dominance task achieves the highest early-exit rate, reaching 56.50%. For samples that proceed to subsequent stages (including EEG processing and ECG–EEG fusion), the computational overhead introduced by the fusion operation is negligible; therefore, these samples are collectively accounted for in the subsequent energy consumption analysis.

The final evaluation demonstrates that, across different emotional dimensions, the average per-sample computational complexity is reduced to 3.70 M (valence), 3.72 M (arousal), and 3.89 M (dominance) FLOPs, with corresponding energy consumption values of 4.54 μJ, 4.60 μJ, and 4.73 μJ, respectively. Compared to the conventional approach in which all samples undergo full-path sequential fusion (“ECG + EEG”), the proposed UDC-SNN framework achieves reductions approximately 50% in both FLOPs and energy consumption. These findings suggest that the adaptive, demand-driven routing strategy effectively suppresses computational redundancy, thereby enabling improved low-power characteristics in multidimensional emotion recognition tasks.

### 4.3. Confidence Threshold Analysis

**Figure 6:**
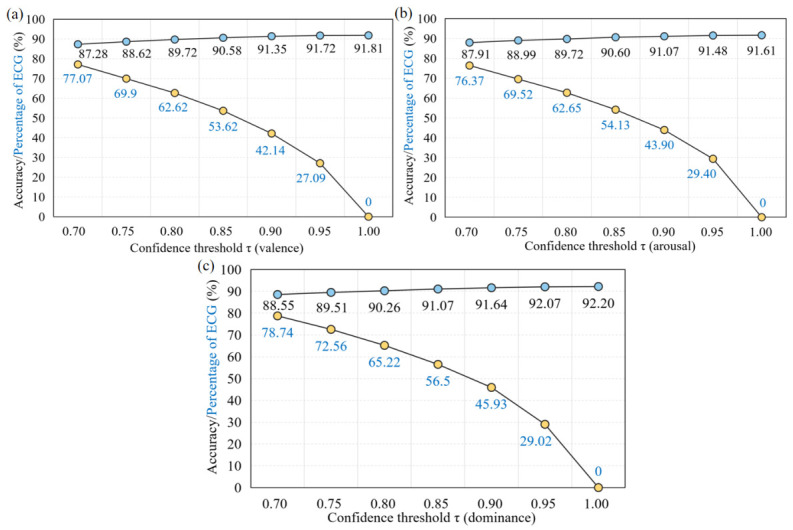
Recognition performance of the UDC-SNN framework for multidimensional emotion recognition under different confidence thresholds and the corresponding proportion of ECG modality utilization. As the confidence threshold varies, (**a**) the valence dimension, (**b**) the arousal dimension, and (**c**) the dominance dimension illustrate the relationship between classification accuracy and the proportion of ECG modality engagement.

This section further investigates the behavior of the UDC-SNN framework under varying confidence thresholds, with a focus on classification accuracy and the proportion of samples processed solely by the ECG modality. The corresponding results are presented in Figure 6.

In conjunction with the energy consumption analysis of ECG and EEG models reported in Table 10, it can be observed that the overall system energy consumption is largely determined by the proportion of samples that complete inference using only the low-cost ECG pathway. This is due to the lower computational complexity of the ECG model compared to the EEG model. The experimental results reveal a clear trade-off between the confidence threshold, system energy efficiency, and classification performance. Across all three emotional dimensions, as the confidence threshold increases monotonically, classification accuracy exhibits a gradual improvement. However, this gain in accuracy comes at the expense of reduced utilization of the low-power ECG pathway, as the proportion of samples relying solely on ECG inference decreases substantially. This indicates that higher accuracy requirements tend to limit the early-exit rate, thereby increasing overall energy consumption.

Moreover, Figure 6 can be interpreted as presenting a continuous sensitivity analysis. By scanning the threshold within the range of 0.7–1.0 with an increment of 0.05, both the overall accuracy and the proportion of samples inferred using the ECG modality exhibit relatively smooth trajectories, without any abrupt or catastrophic fluctuations. This behavior provides supporting evidence for the overall robustness of the proposed system.

Furthermore, the selection of 0.85 is guided by the principle of diminishing marginal returns. Although increasing the threshold (e.g., to 0.9 or 0.95) may yield slight improvements in accuracy, such gains tend to be accompanied by higher computational costs. In particular, when the threshold reaches 1.0, the proportion of samples classified solely by the low-power ECG model drops to zero, forcing all samples to also pass through the complex EEG model, which results in energy consumption that doubles compared to when the threshold is set to 0.85.

Consequently, a threshold of 0.85 emerges as a practical “knee point” for optimization: it maintains accuracy levels above 90% across different emotional dimensions, while avoiding excessive energy expenditure associated with marginal performance improvements.

### 4.4. Comparison with Baseline Configurations

This section systematically evaluates the energy consumption and accuracy of the UDC-SNN framework longside three baseline configurations (i.e., ECG-only, EEG-only, and full ECG + EEG fusion) across different emotional labels (valence, arousal, and dominance), with results summarized in Table 12.

As observed, relying on the ECG-only tends to impose a relatively minimal computational burden, primarily due to the fewer electrode channels involved. Consequently, this mode exhibits low energy consumption across the three dimensions (0.73 μJ, 0.73 μJ, and 0.78 μJ, respectively). However, constrained by limited spatial feature information, its representation capacity is restricted, yielding foundational accuracies of 81.41%, 81.58%, and 83.14%.

In comparison, utilizing the EEG-only captures richer electrophysiological information owing to its increased channel density. This enhances the accuracy across the three dimensions to 88.06%, 88.36%, and 89.10%. Nevertheless, the expanded channel dimensionality inevitably introduces higher computational overhead, raising the corresponding energy consumption to 8.22 μJ, 8.43 μJ, and 9.07 μJ.

Furthermore, adopting the full ECG + EEG fusion scheme (i.e., setting the confidence threshold to 1) allows for the model to fully leverage multimodal complementarity, thereby achieving high accuracies of 91.81%, 91.61%, and 92.20%. However, this performance comes at the cost of energy dissipation caused by the continuous processing of both modalities across all samples, elevating energy metrics to peak levels within this context (8.95 μJ, 9.16 μJ, and 9.85 μJ).

Ultimately, by employing dynamic cascade control over the ECG and EEG modalities, the proposed UDC-SNN framework successfully mitigates computational overhead while preserving high precision. Accuracies are maintained at 90.58%, 90.60%, and 91.07%, showing only marginal deviations from the ECG + EEG mode. Consequently, the framework’s energy consumption is effectively regulated to 4.54 μJ, 4.60 μJ, and 4.73 μJ across the three labels, achieving a reduction of approximately 50% compared to the ECG + EEG baseline.

Beyond energy consumption, real-time performance is also an important criterion for evaluating model feasibility. To this end, we further conducted single-sample inference latency experiments on an NVIDIA RTX 3060 GPU. Each measurement was repeated 100 times, with the warm-up overhead excluded. The corresponding results are presented in Table 13.

The experimental findings indicate that both the ECG model and the EEG model exhibit comparable per-decision latency, on the order of approximately 2 ms. This behavior can be attributed to the fact that, when processing single samples on high-performance hardware such as the RTX 3060, the overall latency is largely constrained by CUDA kernel launch overhead, tensor scheduling, and memory access operations in the SNN, rather than by the computational complexity itself.

Furthermore, the latency of the full ECG + EEG fusion mode is approximately 4 ms. In contrast, the UDC-SNN enables more than half of the samples to be classified directly via the ECG branch, thereby completing the decision process at an earlier stage. As a result, the overall latency falls between that of the ECG model and the full ECG + EEG fusion approach. Consequently, the inference time of UDC-SNN remains substantially lower than the 1000 ms data acquisition window. These results suggest that the UDC-SNN framework achieves high response speeds while maintaining low power consumption.

### 4.5. Comparison with Related Research

To objectively evaluate the contribution of the proposed method, this section presents a comparative analysis with several recent neural network-based approaches in the field. It is worth noting that most existing studies in affective computing primarily focus on classification performance metrics, such as accuracy, while often overlooking the energy consumption associated with large-scale data processing. Consequently, many works do not explicitly report computational complexity (e.g., FLOPs). To ensure both clarity and scientific rigor in comparison, this study adopts a conservative lower-bound estimation approach based on the reported network architectures and key parameters in the literature. Specifically, only the minimum theoretical number of operations involved in forward propagation is considered, excluding non-computational overhead. This provides a relatively unbiased and academically meaningful baseline for comparison. Furthermore, to enhance the comprehensiveness of the evaluation, several state-of-the-art SNN studies are also included in the comparison. The final results are summarized in Table 14.

The work by Siddharth et al. (2022) [20] represents a typical paradigm of leveraging pre-trained visual models for physiological signal analysis.The input signals are transformed into three-channel RGB images and processed using a VGG-16 network pre-trained on ImageNet as the feature extractor. According to the model configuration described in their study, the input image size is fixed at 224×224×3. Based on the standard forward-pass computation of VGG-16, the lower-bound computational cost for processing a single image is approximately 15.3 GFLOPs. In contrast, the proposed method achieves an average recognition accuracy of approximately 90.75% (which is better than the 79.95% reported in that study), while requiring only about 3.77 MFLOPs, which corresponds to roughly $2\times 10^{-4}$ of the computational cost. In terms of energy consumption, the proposed method (4.62 μJ) requires only about $10^{-4}$ of that of the compared approach (35,190 μJ).

The AT2GRU model proposed by Khan et al. (2023) [21] focuses on one-dimensional temporal signal processing and demonstrates relatively favorable computational efficiency. The model complexity is defined as O(gt), where *g* denotes the number of signals and *t* represents the number of time steps. In their DREAMER dataset configuration, the number of hidden units is set to 5 to mitigate overfitting. Based on the standard computational formulation of GRU units, the conservative lower-bound complexity for processing a 1-s sequence sampled at 128 Hz is approximately 2.76 MFLOPs. This places the Khan model in a similar computational scale ($10^{6}$) to the proposed method (3.77 MFLOPs). However, despite its lightweight design, the classification accuracy reported by Khan et al. (84.88%) remains notably lower than that achieved in this study (90.75%). Furthermore, due to the event-driven characteristics of SNNs, the proposed method achieves lower energy consumption (4.62 μJ) compared to their work (6.35 μJ).

More recently, Wang et al. (2025) [22] proposed a hybrid Att-1DCNN-GRU model that enhances feature extraction through high-channel convolutional layers. According to their architectural description, the model employs two one-dimensional convolutional layers (1D-CNN), each with 256 filters and a kernel size of 3. Based on the processing of a 66-dimensional feature vector (comprising 56 EEG features and 10 ECG features), the lower-bound computational cost of the primary convolutional layers is approximately 25.95 MFLOPs. This places the model in the $10^{7}$ complexity range, approximately 7 times higher than that of the proposed method (3.77 MFLOPs). Although Wang et al. report a relatively high accuracy of approximately 95.26% on the DREAMER dataset, the dense filter configuration introduces substantial computational overhead. In terms of energy consumption, the proposed method (4.62 μJ) accounts for only about 8% of the energy consumption in their work (59.69 μJ).

In the context of SNN research, Chen et al. (2025) [28] and Li et al. (2024, 2025) [27,29] have respectively investigated emotion recognition methods based on unimodal EEG signals. To ensure consistency and fairness in the comparison, this study adopts a unified conversion and evaluation framework for both FLOPs and energy consumption, grounded in the single-addition operation characteristic of SNNs and the associated energy cost for ADD operations. The comparative results suggest that the proposed method (90.75%, 4.62 μJ) demonstrates advantages over the approaches of Li et al. (2024: 76.81%, 720 μJ; 2025: 73.11%, 51.21 μJ) in terms of both classification accuracy and energy consumption. although the classification accuracy is slightly lower than that reported by Chen et al. (92.47%, 35.73 μJ), the proposed method achieves an approximate 8-fold improvement in energy efficiency.

In summary, the proposed method achieves a comparatively favorable balance between classification performance and energy efficiency. While maintaining competitive accuracy, it significantly reduces computational and energy costs, thereby offering a more efficient solution for emotion recognition tasks in resource-constrained environments.

It should be noted that, although the comparative results are theoretical references derived from model structure and parameter configurations, this study specifically estimates the lower bound of computational complexity. Since even this lower bound remains inferior to the proposed method, the performance–efficiency advantages of our approach under current task settings remain valid.

## 5. Conclusions

In this work, an uncertainty-aware dynamic cascading framework based on spiking neural networks (UDC-SNN) is proposed for emotion recognition. By incorporating a dynamic routing mechanism with asymmetric computational costs and a Bayesian cascading strategy guided by decision uncertainty, the proposed method improves system efficiency while enhancing the dynamic collaborative representation between ECG and EEG modalities. Experimental results on the multimodal affective dataset DREAMER demonstrate that UDC-SNN performs favorably in terms of both recognition performance and energy consumption. Specifically, this study first investigates the performance of UDC-SNN in terms of accuracy and related metrics, followed by an evaluation of its computational overhead. In addition, ablation studies are conducted to examine its improvements over baseline models, providing evidence for the effectiveness of the proposed framework. Subsequently, the trade-off characteristics of UDC-SNN under different confidence thresholds are analyzed to further understand its dynamic regulation mechanism. Finally, comparative analyses with existing methods suggest that UDC-SNN achieves a meaningful balance between performance and energy consumption, indicating its potential applicability in resource-constrained emotion monitoring scenarios.

While the proposed framework demonstrates promising results, one primary limitation should be acknowledged. The discretization of the original 1–5 self-assessment scale into binary labels, although helpful in mitigating noise in subjective reports, may inevitably lead to a loss of fine-grained affective information.

In future work, we plan to explore more advanced modeling strategies to enable fine-grained emotion recognition. In addition, we will consider deploying the UDC-SNN framework on neuromorphic hardware platforms such as Intel Loihi. This may involve low-bit quantization and topology-aware mapping, combined with confidence-threshold-triggered dynamic power-gating mechanisms, with the aim of further improving the efficiency of physiological signal processing.

## Figures and Tables

**Table 1:** Data profile after segmentation.

Type	Recordings	Time Length (s)	Sampling Frequency (Hz)
Valence ≤ 3.0 (low)	100,518	1	128
Valence > 3.0 (high)	70,142	1	128
Arousal ≤ 3.0 (low)	88,844	1	128
Arousal > 3.0 (high)	81,816	1	128
Dominance ≤ 3.0 (low)	78,964	1	128
Dominance > 3.0 (high)	91,696	1	128

**Table 2:** Configuration of key parameters for ECG and EEG models.

Module	ECG	EEG
Stage 1	Conv (3×7,1×2,1×3) Pool (1×2,1×2)	Same as ECG
Stage 2	Conv (3×5,1×2,1×2) Pool (1×2,1×2)	Conv (3×5,1×2,1×2) Pool (2×2,2×2)
Stage 3	Conv (3×3,1×1,1×1) Pool (2×2,2×2)	Same as ECG
Loss	Smooth Factor =0.05	Smooth Factor =0.1

**Table 3:** Classification performance of ECG and EEG model under different labels.

Model	Acc (%)	F1 (%)	Pre (%)	Rec (%)	Spe (%)	AUC (%)
ECG-valence	81.41	77.14	77.96	76.35	84.94	89.19
ECG-arousal	81.58	81.01	80.07	81.97	81.22	90.20
ECG-dominance	83.14	84.53	83.38	85.70	80.17	91.01
EEG-valence	88.06	85.23	86.73	83.78	91.06	95.13
EEG-arousal	88.36	87.76	88.47	87.06	89.56	95.59
EEG-dominance	89.10	89.79	90.41	89.17	89.02	95.84

**Table 4:** Classification performance of ECG model under different labels at different time steps.

T	Acc (%)	F1 (%)	Pre (%)	Rec (%)	Spe (%)	AUC (%)
T3-valence	79.95	75.08	76.75	73.48	84.47	87.76
T3-arousal	80.64	80.13	78.88	81.41	79.93	89.26
T3-dominance	82.51	84.06	82.37	85.82	78.66	90.44
T4-valence (Ours)	81.41	77.14	77.96	76.35	84.94	89.19
T4-arousal (Ours)	81.58	81.01	80.07	81.97	81.22	90.20
T4-dominance (Ours)	83.14	84.53	83.38	85.70	80.17	91.01
T5-valence	80.72	76.12	77.51	74.78	84.86	88.85
T5-arousal	81.68	81.14	80.09	82.21	81.18	90.42
T5-dominance	83.56	84.87	83.92	85.85	80.90	91.45

**Table 5:** Classification performance of EEG model under different labels at different time steps.

T	Acc (%)	F1 (%)	Pre (%)	Rec (%)	Spe (%)	AUC (%)
T3-valence	87.02	83.86	85.71	82.09	90.45	94.45
T3-arousal	87.06	86.41	87.05	85.77	88.25	94.58
T3-dominance	85.77	86.65	87.37	85.93	85.57	93.88
T4-valence (Ours)	88.06	85.23	86.73	83.78	91.06	95.13
T4-arousal (Ours)	88.36	87.76	88.47	87.06	89.56	95.59
T4-dominance (Ours)	89.10	89.79	90.41	89.17	89.02	95.84
T5-valence	87.71	84.85	85.97	83.76	90.46	94.76
T5-arousal	88.29	87.75	87.98	87.52	88.99	95.40
T5-dominance	88.64	89.42	89.46	89.39	87.77	95.52

**Table 6:** Classification performance of UDC-SNN under different labels.

Label	Acc (%)	F1 (%)	Pre (%)	Rec (%)	Spe (%)	AUC (%)
valence	90.58	88.34	89.96	86.77	93.25	95.43
arousal	90.60	90.19	90.23	90.14	91.02	95.99
dominance	91.07	91.75	91.06	92.45	89.46	95.81

**Table 7:** The accuracy of the models and framework on the Amigos dataset.

Label	Acc (%)		
	ECG	EEG	UDC-SNN
valence	68.49	66.07	71.16
arousal	57.89	61.36	63.31
dominance	62.85	64.65	68.68

**Table 8:** Energy costs of addition and multiplication in 45 nm CMOS (32 bit).

FP ADD	FP MULT	FP MAC
0.9 pJ	3.7 pJ	0.9 + 3.7 = 4.6 pJ

**Table 9:** Energy consumption improvement of the ECG/EEG model over the isomorphic ANN model under different labels.

Model	Normalized ${\mathrm{OP}}_{\mathrm{ANN}}$ (*a*)	Normalized ${\mathrm{OP}}_{\mathrm{SNN}}$ (*b*)	OP ${\mathrm{Layer}}_{\mathrm{ns}}$/ Total ${\mathrm{OP}}_{\mathrm{ANN}}$ (*c*)	ANN/SNN Energy $\left(\frac{a\times 4.6}{c\times 4.6+(1-c)\times b\times 0.9}\right)$
ECG-valence	1.0	0.14	0.04	15.08
ECG-arousal	1.0	0.14	0.04	15.08
ECG-dominance	1.0	0.16	0.04	14.28
EEG-valence	1.0	0.47	0.06	6.83
EEG-arousal	1.0	0.48	0.06	6.74
EEG-dominance	1.0	0.54	0.06	6.28

OP ${\mathrm{layer}}_{ns}$: The number of non-impulsive operands in the model.

**Table 10:** Energy consumption of ECG/EEG model under different labels.

Model	Parameters (M)	FLOPs (M)	Energy (μJ)
ECG-valence	0.24	0.52	0.73
ECG-arousal	0.24	0.52	0.73
ECG-dominance	0.24	0.56	0.78
EEG-valence	0.50	6.86	8.22
EEG-arousal	0.50	6.97	8.43
EEG-dominance	0.50	7.66	9.07

**Table 11:** Energy consumption of UDC-SNN under different labels.

Label	UDC-SNN				ECG + EEG	
	Sample Size (ECG)	Sample Size (ECG + EEG)	FLOPs (M)	Energy (μJ)	FLOPs (M)	Energy (μJ)
valence	9150 (53.62%)	7916 (46.38%)	3.70	4.54	7.38	8.95
arousal	9238 (54.13%)	7828 (45.87%)	3.72	4.60	7.49	9.16
dominance	9642 (56.50%)	7424 (43.50%)	3.89	4.73	8.22	9.85

**Table 12:** Comparison of UDC-SNN with the baseline in terms of energy consumption and accuracy across different labels.

Label	ECG		EEG		ECG + EEG		UDC-SNN	
	Acc (%)	Energy (μJ)	Acc (%)	Energy (μJ)	Acc (%)	Energy (μJ)	Acc (%)	Energy (μJ)
valence	81.41	0.73	88.06	8.22	91.81	8.95	90.58	4.54
arousal	81.58	0.73	88.36	8.43	91.61	9.16	90.60	4.60
dominance	83.14	0.78	89.10	9.07	92.20	9.85	91.07	4.73

**Table 13:** Comparison of UDC-SNN with the baseline in terms of latency across different labels.

Label	Latency (ms)			
	ECG	EEG	ECG + EEG	UDC-SNN
valence	1.75±0.26	1.84±0.31	3.83±0.36	1.75±0.26∼3.83±0.36
arousal	1.76±0.34	1.85±0.29	3.82±0.43	1.76±0.34∼3.82±0.43
dominance	1.75±0.24	1.92±0.37	3.88±0.46	1.75±0.24∼3.88±0.46

**Table 14:** Comparison of the performance and energy consumption in related research.

Literature	Signal	Acc (%) (Valence/Arousal/ Dominance)	FLOPs (M) (Valence/Arousal/ Dominance)	Energy (μJ) (Valence/Arousal/ Dominance)
[20]/2022	ECG + EEG	79.95/79.95/- (Average = 79.95)	∼15,300	∼35,190
[21]/2023	ECG + EEG	84.15/84.69/85.81 (Average = 84.88)	∼2.76	∼6.35
[22]/2025	ECG + EEG	95.95/94.93/94.91 (Average = 95.26)	∼25.95	∼59.69
[27]/2024(SNN)	EEG	71.01/78.50/80.92 (Average = 76.81)	∼800	∼720
[28]/2025(SNN)	EEG	89.82/93.69/93.90 (Average = 92.47)	∼39.7	∼35.73
[29]/2025(SNN)	EEG	64.01/76.33/78.99 (Average = 73.11)	∼56.9	∼51.21
**Ours**	ECG + EEG	90.58/90.60/91.07 (Average = 90.75)	3.70/3.72/3.89 (Average = 3.77)	4.54/4.60/4.73 (Average = 4.62)

## Data Availability

Data are contained within the article.
